# Anti-doping sciences, abjection and women’s sport as a protected category

**DOI:** 10.3389/fspor.2023.1106446

**Published:** 2023-05-24

**Authors:** Angela J. Schneider, Alan C. Oldham, Loughran H. G. Butcher

**Affiliations:** ^1^ International Centre for Olympic Studies, Western University, London, ON, Canada; ^2^ Faculty of Health Sciences, School of Kinesiology, Western University, London, ON, Canada; ^3^ Ethics and Public Affairs, Carleton University, Ottawa, ON, Canada

**Keywords:** anti-doping science, abjection, women's sport, abjection bias, gender diverse athletes, abjection potential, intersectional abjection

## Abstract

In this article we explore the relationships amongst anti-doping sciences, ‘abjection,’ and the protection of ‘women's’ sport. We introduce three novel concepts: ‘abjection bias,’ ‘abjection potential,’ and ‘intersectional abjection,’ as tools with the potential to provide greater nuance to understanding the context for these contentious issues in contemporary sport. The debate concerning participation in women's sport—especially elite sport—of people who do not fit within traditional definition of ‘women’ is increasingly fraught with acrimony with anti-doping sciences often recruited as arbitrator. With access to opportunities such as participation at the Olympic Games at stake, emotions run high in arguments that typically centre on inclusion of transgender and gender diverse (TGD) athletes on the one hand and protection of the women's category on the other. While sport theorists have begun the important work of identifying the roots of these problems deep within the structure of modern sport and society itself, they have hitherto paid little attention to the philosophical underpinnings of that structure. Through the lens of feminist critical analysis, we seek, in this paper, to understand the complex role of ‘abjection’ in framing the current debate in sport and in related anti-doping sciences. From a clear definition of abjection as a perceived existential threat due to violation of the status quo, we introduce the new concepts of ‘abjection bias,’ ‘abjection potential,’ and ‘intersectional abjection’ in order to understand and explain what in common parlance we might call ‘gut reaction.’ By looking at the few notable previous treatments of sport abjection and highlighting the historical connections between anti-doping sciences and efforts to protect the women's category, we demonstrate that this co-development is, in part, more easily understood in the context of ‘abjection.’ We conclude that the clarity gained can also help to shed light on current policy decision-making in relation to the question of protecting the women's sport category.

## 1. Introduction

In this article we explore the relationships amongst anti-doping sciences, “abjection,” and the protection of “women’s” sport. These main ideas will be introduced thematically in the following sections. We begin in section 2 by examining the concept of “abjection” as a response to perceived existential threats to the *status quo*. We propose the idea of “abjection bias” as a conceptual tool for understanding how abjection arises. In section 3, we establish the connection between “abjection” and the “Unnaturalness Argument,” itself analogous to the “Frankenstein factor” from bioethics, and responses to it, through the development and application of anti-doping sciences. In section 4, we look at applications of abjection theory to sport. First, we engage with David Fairchild's application of abjection theory to the topic of doping in sport through the case of Ben Johnson, disgraced Canadian sprinter stripped of Olympic gold after a positive doping test at the Seoul 1988 Olympics. While Fairchild's argument was important as the first to identify abjection in sport, we reject his dangerous suggestion that popular sentiment, the “yuk” factor, might guide anti-doping policy and practice—what we label an “abjection test.” Then we engage with Kutte Jönsson's use of abjection theory in the context of (dis)ability in sport. Jönsson identifies a double standard in abjection potential between able-bodied and (dis)able-bodied athletes premised on a pre-existing abjection of (dis)ability. Notably, while the topic of abjection is largely underutilized in sport, Fairchild and Jönsson are not alone; Michael D. Burke's and Terrence J. Roberts' significant 1997 look at the role of abjection in perceptions of female athletes using performance enhancing drugs and Burke's 2001 PhD thesis examining the female athlete as abject are clear demonstration of the applicability of the concept within the field of sport philosophy.

It is our aim here not simply to apply the concept to anti-doping sciences and the protected category of women's sport but to also introduce the novel concepts of “abjection bias,” “abjection potential” and “intersectional abjection” that allow for a critical weaving together the various threads presented by Fairchild's (doping) and Jönsson's ((dis)ability sport) applications within a larger framework shedding light on the broad phenomenon of abjection in sport. Linking this with the idea of abjection bias, we posit that Jönsson's double standard is at work more broadly as an example of the tendency to abject what is perceived to be “unnatural”, which can include female athletes relative to normative “male” athletes, as well as transgender and gender diverse (TGD) athletes relative to “natural” biological females. In section 5, we explore the history and context of women as a protected category in sport. Section 6 includes some ethical concerns that must be addressed in the attempt to overcome abjection in the development and implementation of policy regarding the pressing issues of doping and TGD athlete participation in the “women’s” protected category. We conclude that anti-doping sciences have been used in part in attempts to protect and maintain the category of women's sport and that the concept of abjection can be applied both to doping and perceived violations of sport's traditional gender binary. Our increased understanding of the potential role that the concept of “abjection” has, and continues to play, in these two applications of anti-doping sciences gives us a more comprehensive perspective of this complex human reaction. The clarity gained from understanding this reasoning can also help to shed light on current policy decision-making as well as public and political dialogue regarding the protection of the ‘women's category in sport.

## 2. Abjection and the “abjection bias”

With its roots in psychoanalytical and (post) structuralist theory, abjection was extensively developed by Bulgarian born French philosopher Julia Kristeva in her influential 1980 essay, “Povoirs de l”horreur: Essai sur l”abjection.”^1^ Kristeva's work has frequently been engaged with through the broad lens of critical theory and particularly in film analysis.^2^ For Kristeva, abjection constitutes an absolute rejection of something or someone who appears to violate in some extreme way the established order of things.^3^

It is thus not lack of cleanliness or health that causes abjection but what disturbs identity, system, order. What does not respect boarders, positions, rules. The in-between, the ambiguous, the composite. The traitor, the liar, the criminal with a good conscience, the shameless rapist, the killer who claims he is a savior. [… Abjection is] immoral, sinister, scheming, and shady (1).

Building on this idea, theorist Barbara Creed wrote that,

The place of the abject is where meaning collapses, the place where I am not. The abject threatens life, it must be radically excluded from the place of the living subject, propelled away from the body and deposited on the other side of an imaginary border which separates the self from that which threatens the self (2).

Abjection, therefore, “draws our attention to the place where meaning collapses”, an unknown in-between state of the known (3). As such, we reject the abject from ourselves. That which is abjected (the abject^4^) is situated^5^ in a no-place^6^ beyond the symbolic order that holds together. The abject cannot—indeed must not—be engaged with beyond the most fundamental instinct that it must be repulsed lest the self be consumed, tainted, collapse into the oblivion of abjection even simply through association with, or contemplation of the abject.

As for the theoretically uncomfortable fact that not everyone experiences feelings of abjection in similar situations, Robbie Duschinsky, in particular, positions Kristeva's “abjection” within a more nuanced relationship with the status quo.

Not all phenomena that we classify as impure is in-between or ambiguous and not all in-between or ambiguous phenomena are impure. Rather […] the impure is that which is constructed as deviating from an essential state of original homogeneity (4). Duschinsky builds on this, pointing to Kristeva's idea of abjection as a perceived violation of integrity. “The breach of integrity,” states Duschinsky, “does not primarily invoke purity and impurity by virtue of an ambiguity between self and other, but through evoking an image of the contamination of a prior homogeneity by the intrusion of heterogeneity” (4).

This idea offers a clear approach to the application of abjection theory to sport. In this attempt, we can also look to Duschinsky's own “take” on impurity, that “in Western societies, impurity characterizes by degrees all phenomena that deviate from what is imputed as their *self-identity:* their internal homogeneity and their correspondence with elsewhere.” Qualifying this, Duschinsky suggests that

Western assumptions about essence as a state of internal homogeneity underpinning existent phenomena are shaped by a particular cultural heritage. Purity and impurity, as appeals to self-identity, appear in discourses as diverse as those on the body, sexuality, political corruption, nationalism, waste and rubbish—wherever a qualitatively homogenous essence is taken to underpin existence (4).

With such a framework in mind, in this paper we argue that abjection is at work in official and popular perceptions of, and reactions to, contemporary sport's two most significant issues: doping and questions of inclusion in sex/gender categories. In both situations, abjection seems to arise where violations of a perceived homeostasis precipitates existential crisis. Thus, it may well be asked what role abjection plays in decision making processes designed to protect the status quo in the face of cheaters who dope, break the rules and manipulate their bodies, (dis)able-bodied athletes, women in general, and to those challenging the gender dichotomy within sports.

For conceptual clarity, we propose a new term “abjection bias” to describe what might less accurately be called “gut reaction” in what should be—and ethically must be—a rational process. We suggest the following definition: *the abjection bias occurs when feelings of abjection about an individual, group, action, situation* etc. *uncritically guide the decision-making process. Decisions influenced by the abjection bias might be identified by their reliance on rhetorical, impassioned appeals to a shared sense of disgust/revulsion in order to create metaphorical or real distance between a person's identified group and that which gives rise to the feeling of abjection*.

In this paper we will argue that the abjection bias has played a significant part in shaping responses to perceived threats against “clean sport” and “women’s sport.” The analysis of the concept of “abjection,” and identifying the role of abjection bias, deepens our understanding of responses defending the perceived *status quo*. Indeed, as we explore the nature of abjection and become aware of an abjection bias, we can begin to hope for rational and perhaps novel approaches to disentangling the Gordian knot of contentious issues such the inclusion/exclusion of TGD athletes and the protection of women's sport. Further, we suggest that the idea of abjection bias can help to explain and challenge what some sport philosophers have called the “Unnaturalness argument,”^7^ to be explored in the following section.

## 3. Abjection, the “Frankenstein factor” and the “unnaturalness argument” as used in the anti-doping sciences

Abjection correlates well to the “unnaturalness argument” often put forward that doping is bad because there is something “unnatural” about it.^8^ Yet the unnaturalness argument is problematic since it is premised on an unprovable first principle, the nature of what is “natural.” Sport philosopher Roger Gardner's description of the “natural” illustrates well this limitation:

*Any procedures that might* change *or control `the nature of our species'* [*…*] *somehow threaten `our sense of identity, our sense of uniqueness* […]*’* [*…*] *\such prospects threaten wholly to subvert traditional philosophical paradigms and undermine the standard ethical touchstones of `human nature,' `humanity,' and `rationality;’* […] (5).

As with the analogous “Frankenstein factor,” a binary distinction between “natural” and “unnatural” opens the door to abjection bias for anything or anyone threatening the symbolic order of the status quo.^9^ The similarities between “abjection” and the “Frankenstein Factor” from the bio-ethics literature is striking and has not been discussed in the literature before. Even as Kristeva was formulating her theory of abjection in the 1970s, Willard Gaylin's used the term “Frankenstein Factor,” to describe societal fears surrounding extraordinary new research with significant impact on human beings. For many issues including drug use or DNA research in general, Gaylin proposed it as “an unanalyzed element coloring the debate,” (7). This is also analogous to the “yuk” factor, as a metaphor for the physical response that coincides with the recognition of “extreme other.”

Historically, there are many ways in which the “unnaturalness argument” has been used in support of anti-doping sciences (5) but it is fair to draw a conclusion that the argument itself is flawed. If we had a sound and consistently used definition of “unnatural” in this context, then perhaps the unnaturalness of those substances, methods, or amounts found to be such could be used to define the practices which are deemed wrong. The fact, however, is that we do not.

Despite these challenges to the various articulations of the “unnaturalness argument,” justification for prohibiting substances can and has differed from substance to substance and might even shift as both science and opinions change over time. Thus, some substances might have been considered undesirable due to the mode of introduction in some cases and in others because of the artificial nature of the substance itself, and in still others for reasons beyond the scope of this paper such as risk of harm or violations of the “spirit of sport.”

Regardless of the justification of a banned substance, the result and, perhaps most of all, the intent to exceed pre-existing limitations and perceptions of what is humanly possible are perhaps most central to the question of abjection bias and the “Unnaturalness argument.” Gardner captures this idea well, saying that,

the gained enhancement is viewed to be beyond the athlete's human capabilities. It would seem that we are opposed to such enhancement because […] we wish to view the athletes as the counterparts not of gods but of demigods. This is conditioned by the ambiguous character of their deeds which we wish to view as superhuman but definitely not nonhuman. Elite athletes exceed what average human beings are capable of achieving […] It is along these lines that we may wish to argue against substance-acquired capabilities: they permit athletes to transcend the boundary of humanness (5).

Thus, “natural” and “unnatural” attempts to mean what is “natural” or “unnatural” for us as human beings. The primary concept in terms of which “natural” had to be defined was that of a human or a person. If we do not have a consistent view of what it was to be human, we cannot define what is natural or unnatural. Deciding what it is to be human is logically prior to determining what is, or is not, an “unnatural’ practice.

However, at the intuitive level these arguments struck a chord, particularly for the view of female athletes using steroids. These arguments were based on the claim that the banned practice threatened the essence of the athlete's humanity, and for women, it was their “womanhood” that was threatened. As mentioned above, in the bioethics literature this idea fits well with Willard Gaylin's terminology of the “Frankenstein Factor”, used to describe societal fears of the effects of things like bio- technology and drugs in general (6). The “Frankenstein Factor” theme is related to the “artificial” construction (or manipulation) of a hybrid human being.^10^ For female athletes, the response is usually negative and often worse, because it was viewed as turning them into men. This response was highlighted in early anti-doping educational materials. In the case of its application to the athlete, doping is viewed manipulating them beyond a natural human being.

Thus, although the “Unnaturalness Argument” has been robustly challenged on many fronts, it has persisted as a central rationale in anti-doping policy and messaging. We propose that abjection is at least in part responsible.

It is important to note, that such criticisms of the “Unnaturalness Argument” are not to argue in favour of doping, but rather to point out first, the inherent weakness of the “Unnaturalness argument” and second, despite this logical fallacy, that any distinction between “natural” and “unnatural” strengthens abjection bias against the latter. For these reasons, officially sanctioned/leveraged abjection of doping or dopers—as we shall see in the next section with Ben Johnson—has been a convenient expedient in the fight not just to protect “clean” sport,^11^ but also to protect “women's” sport.^12^

## 4. Sport abjection: Fairchild on doping, Jönsson on (Dis)ability sport, “abjection potential,” and “intersectional abjection”

### 4.1. Fairchild's “abjection test” rejected

David Fairchild was the first to apply the concept of abjection to sport in his 1989 paper “Sport Abjection: Steroids and the Uglification of the Athlete.” Fairchild focused on the fall from grace of Canadian sprinter Ben Johnson to argue that the idea of “abjection” can help us understand our (over) reactions towards doping in sport, a practice that challenges prevailing notions of order and purity. “Such an understanding,” he suggested, “may enhance the possibility of developing rationally defensible policies governing the use of such substances and practices” (7).

While this objective seems to resonate with our own, we reject Fairchild's conclusion that abjection might serve as a barometer of public sentiment to direct policy concerning right and wrong behaviors.

This essay has suggested that the concept of abjection may illuminate certain issues arising from substance abuse in sport, perhaps including an appreciation of argument types appropriate for justifying prohibitions on substance use. Specifically, justifications for regulating substances must proceed from the recognition that the use of most substances for which prohibitions are sought generates common, vigorous, public disapproval (7).

In appealing to “common sense,” Fairchild's “abjection test” continues in a long history of *a priori* argumentation positioning as “natural” the *status quo* and as “unnatural” challenges to that established order. In other words, fertile ground for abjection bias to run amok, as shown in the previous section's overview of the “Unnaturalness argument” and its (mis)uses in anti-doping theory. Thus Fairchild's “abjection test,” in its reliance on the “Unnaturalness argument,” must be rejected in favour of a more rational and nuanced understanding.^13^ Even though Fairchild's conclusion is flawed—as we demonstrate below—he presents a good example of the “mechanics” of sport abjection in his analysis of Ben Johnson. Fairchild tries to explain the logic of doping-related abjection through i) the boundary of the body; and ii) the limits of the body. For the boundary of the body, he makes an inner/outer distinction, where things like “spittle, blood, milk, feces, urine or tears” (7), once they have been passed, cannot enter the body without abjection. “The deliberate reinsertion into the body, through ingestion or injection, of substances that have traversed the body”s boundaries is both an abrogation of the fundamental inner/outer distinction that determines our own clean selves and a culturally revolting practice” (7).

Abjection is worse for athletes because they are exemplars, with whom we identify. “Their participation in certain culturally revolting behaviours leads to an especially dramatic form of “abjectification” (7). Significantly, Fairchild also points to a broader social context within which abjection is wielded as a means of separating clean from unclean, citing Kristeva”s phrase “the simple logic of excluding filth” (7).

While Fairchild can be justly criticized for his reliance on the “Unnaturalness argument,” we suggest that his work's significance lies primarily in the connection of the concept of abjection to the official and popular reaction to Johnson's doping revelations. To put things into perspective, Fairchild was writing at a time immediately following what has more recently been called “the dirtiest race in history” (8). Johnson had stunned the world with his incredible performances and captured Gold in the 100 meter final at the Seoul 1988 Olympic Summer Games. Yet in the very moment of glory he tested positive for a banned performance enhancer. Johnson had doped, and what's more, once caught, he admitted to years of systematic drug use to gain a competitive advantage.^14^

Following his admission, Johnson was banned from further Olympic competition, suspended from his national team, and in September, 1989 stripped by the International Amateur Athletic Federation (IAAF) of various titles, medals won, and records set in non-Olympic competition dating back to 1981 (7).

Many, including Fairchild, were trying to make sense of what had just happened. “In the span of just a few months,” he wrote to introduce the concept of abjection, “Johnson has lost more than just his stature as a body beautiful. Our initial fascination has turned to revulsion, and he has been declared a track nonperson. We have abjectified him” (7).

Fairchild's idea of applying abjection to sport is picked up by Burke and Roberts in 1997, who similarly apply it to the case of doping in sport, but specifically in relation to women. One major contribution that they make, and one that further cements the links between Fairchild's application of abjection and that of |Jönsson (2017) explored just below, is in positioning the source of abjection within a broader societal context.

Whereas Fairchild suggests that the legislation banning drug use is driven by our, that is, the sport community's, revulsion of the drug user's *athletic* body, we will suggest that at least part of the force behind the drug ban is due to our abhorrence of the drug user's social body (8).This understanding of athletes as embodying identities beyond sport (*e.g.,* sexed bodies, gendered bodies, racialized bodies, (dis)abled bodies, in other words “socially constructed bodies”), opens up the door to two concepts that we will develop in the remainder of this section: “abjection potential” and “intersectional abjection”. *Jönsson's “double standard” acknowledged, and two new concepts proposed: “abjection potential” and “intersectional abjection”*

After Fairchild (7), Burke and Roberts (8), and Burke (9), Kutte Jönsson (10) provides what might be the only other sustained academic application of abjection theory to sport. While Fairchild looks at abjection through the lens of the boundary-transgressive, “unnaturalness” of injecting performance enhancing substances into the body to explain the abjectification of Canadian sprinter Ben Johnson, Jönsson focuses instead on the subject's inability to fully remove themself from the threat of the abject in the form of the (dis)abled elite athlete. His 2017 work on abjection and parasport is significant in the introduction of the idea of a double standard in how we experience abjection in relation to able-bodied versus (dis)able-bodied athletes.

By framing his discussion of the term “freak show” as a description of the non-normative, unnatural ((dis)abled) bodies of Paralympians, Jönsson applies the idea of abjection to Paralympians and Olympians alike as similarly disturbing what can be considered a “normal” human.

In relation to sports, and elite sports in particular, there is another point to make in regards to this, something that has to do with the irony of elite sports. And that is that elite sport *in itself* may be seen as a producer (and not just a container) of abjects. In many ways one can claim that elite athletes in themselves, with or without taking performance enhancing drugs (as in the case of steroid users), they become “abnormal” according to a social constructivist view (11); Jönsson's emphasis).

Yet, drawing a cultural distinction between perceptions of able-bodied and (dis)able-bodied individuals, Jönsson acknowledges a double standard, that “as long as [able-bodied] athletes follow “the rules,” and as long as they do not challenge the commonly defined notion of good taste, they will never be classified or diagnosed as abjects […] Can the same be said about current Paralympians? Probably not” (11). The different standard to which athletes of diverse abilities are held in the popular imagination is also evident in their being viewed and discussed as (dis)abled first and elite athletes second.^15^

Jönsson's identification of this double standard is important in analytically separating abjectionable behaviour (*e.g*., “freakishly” exceptional athletic ability) performed within the rules by otherwise “normal” individuals (*e.g.,* able-bodied elite athletes) and similar behaviour performed by individuals who are in some way not considered “normal” (*e.g.,* a Paralympic gold medalist). In these examples, following Jönsson's rationale, we are unlikely to consider the Olympic gold medalist abject, but we may well—are even likely to—consider as abject the Paralympic gold medalist standing on the same podium just a few weeks later.

For Jönsson this comes back to “an established conception of what “bodily perfection” means, a conception that seems to be deeply imbedded not least within the ideology of the Olympics” (11). “[W]hen it comes to able-bodied athletes we usually tend to see the freakishness of outstanding performances as something admirable in itself” (11).

Jönsson concludes that the “Paralympics represent something different from the Olympics. In other words, it seems much easier to connect disability sport to the term freak show [and, we might add, feelings of abjection] than able-bodied sports” (11). As abjects, society seems content that these athletes simply be able to compete against each other. Yet, attempts to bridge the divide between (dis)able-bodied and able-bodied sport reveal the extent of abjection bias against para-athletes. Take the famous example of South African Paralympic champion, Oscar Pistorius, who lobbied to compete in the “able bodied” Olympic Games. Despite a level of abjection due to his circumstances as a para-athlete, the unnaturalness, particularly of his prosthetic legs, can be viewed as central to his further abjection. He was ultimately denied the chance to race with the fastest (able-bodied) Olympians for arguments that were also, in part, based on fair play and competitive advantage, the reaction in some ways parallels Ben Johnson's. For Pistorius, what we accepted and celebrated (the use of the prosthetic Cheetah blades for example, in the “freakshow” Paralympic context where “freaks“ (abjects) are merely competing against other “freaks“ and “tolerably” abject on that grounds) was transformed into something that we revile as “unnatural” on an entirely different level when that same “freak of nature” seeks to use his “unnatural”, “artificial”, “Frankenstein” adaptations to compete with a “normal” human. “As Jönsson points out, elite athletes are never really “normal”. Thus, Usain Bolt for example, while theoretically abjectionable on the grounds that he was the fastest human in the world at that time, yet as he was perceived to be following the rules, he retains his “body beautiful” status. Pistorius’ abjection becomes all the greater by daring to challenge that “natural” human ability. This link is further strengthened by the moniker “technology doping” that is often applied to the phenomenon of creating an unfair or “unnatural” advantage through ability-improving para-sport technology such as Pistorius’ “cheetah blade” prosthetic legs. The case is similar for TGD athletes, whose biological and/or hormonal “advantages” are conflated with doping.^16^

Taking this one step further we suggest that all behaviours or characteristics that challenge the *status quo* possess a certain “abjection potential.” The activation or realization of this potential, to a greater or lesser degree, depends on the circumstances of the individual or group engaged in the behaviour or action. Further, we propose that the idea of individual or group circumstances, or intersecting identities, impacting an individual's experience of abjection, or likelihood of abjection, also opens the door to a theory of “intersectional abjection.”^17^

These concepts help us to identify abjection bias stemming from pre-conceived notions of what constitutes “normal” and hence desirable. “We still consider the athletes with artificial limbs to be essentially undesired,” states Jönsson, “in that they still will be representing a dimension in the human condition most people consider to be connected to emotions of horror” (11). Once again, the connection to the bioethics' “Frankenstein factor” here are clear. We also suggest the presence of abjection bias as a major factor in the implicit acceptance of the unnaturalness argument.

As we will demonstrate in the next section, it is not just doping or (dis)ability that elicits feelings of abjection in this way. Abjection bias, and with it Jönsson's double standard, can be identified in the debate about what constitutes a “woman” for the purposes of sport. Individuals who do not conform to prevailing cultural attitudes about sex and gender are, by their very (perceived) unnaturalness, sites of abjection. Thus, just as for persons with (dis)abled bodies, individuals transgressing sex/gender norms already face abjection bias and that pre-existing bias seems to activate elite sport's abject potential in a way similar to elite athletes who dope.

## 5. Context for women's sport as a protected category

Before looking more specifically at the potential abjection bias's role in official and public abjection of women, transgender and gender diverse (TGD) athletes, it is important to understand the history of the complexity of the establishment and maintenance of women's sport as a protected category. The connections between anti-doping sciences and the “protection” of women's sport are numerous; this has frequently led to the conflation of protecting “women's” sport with protecting “clean” sport, a distinction obscured further by abjection bias. The clarity that can be gained from understanding this history can help to shed light on particular aspects of current policy decision-making.

The debate about women's place in competitive sport has roots in antiquity where women were not permitted to take part or even watch the Olympic Games. Two millennia on, at the first modern Olympic Games at Athens 1896, although women could spectate, they were similarly barred from competing. As things changed—slowly—with the inclusion of some women's and even mixed events at Paris 1900, the debate around policing who was, and who was not, a woman for the purposes of sport began in earnest.

Researchers, who for the most part were men, —from the bourgeoning fields of biological science and medicine, were prominent voices in discussions on the “protection” of women's sport taking place within the IOC and other governing bodies of international sport in the early 20th Century (12). Policies linked to practical action to protect women's sport started in the late 1930s. The creation of these policies and practices coincided with the views of Avery Brundage, President of the IOC from 1952 to 1972. Raising sex verification concerns in a 1936 letter addressed to Henri de Baillet-Latour, then IOC President, Brundage, then head of the American Olympic Association (precursor to the United States Olympic Committee), wrote,

[…] *I do know that the question of the eligibility of various female (?) athletes in several sports has been raised because of the apparent characteristics of the opposite sex. Recently considerable publicity was given* […] *to the case of an English athlete who after several years of competition as a girl announced herself (?) to be a boy* (13).

This position is important to note for the purposes of this discussion for two reasons. One, it is clear that Brundage, whose voice was an increasingly powerful one within the Olympic movement, viewed such activity as a form of cheating, similar to doping. The second reason is that, historically, connections between doping and sex verification, in particular specific comments by Brundage, seem to combine two distinct claims: first, a concern about masculinization of female athletes; and second, a concern about male athletes pretending to be female.

It has been well established that in Brundage's tenure at the IOC's helm, beliefs about female athletes and sport were often conflated with, and confused by, societal concerns about sport participation causing masculinization (14). This confusion has contributed to complicating attempts, even now, to assess the need for protection of the women's category in sport. As it happened, Brundage was concerned enough to recommend,

[…] *that all women athletes entered in the Olympics be subjected to a thorough physical examination to make sure they were 100% female* […as] *athletes who recently competed in European track events as women were later transformed into men* (15).^18^

Responding to the concern of protecting women's sport, the International Association of Athletic Federations (now World Athletics) created a formal sex verification process in 1937. There is, however, some evidence of “physical examinations” before this date (16). This new rule was implemented in the “protests” section of the IAAF policy, where it states that, if the “protest concerns questions of a physical nature, […] physical inspection be made by a medical expert” (17). Thus, the decision-making and justificatory power fell upon the experts from the medical/biological sciences.

Significantly the authority over sex verification remains to this day the remit of medical “sport sciences.” Given the increasing role granted to these same medical experts by the IOC and IAAF to deal with the threat of doping, beginning in the 1950s and 60s (12), the confusion and conflation of these two issues is unsurprising. As the issue of sex verification progressed, the governing bodies of sports shaping anti-doping and sex testing policies did not address complex ethical concerns creating obstacles in the pursuit of relying purely on medical science to determine what constitutes “natural” sex/gender (12). The pursuit of sex verification through medical science was catalyzed by the entrance of the Soviet Union (USSR) in the Olympic Games in 1952; not only raising geopolitical power struggles in international sport, but also, at the same time, presenting dominating, powerful and muscular Soviet women in sport. Concern was soon noted by IOC and related officials, that not only were the Soviets using athletes doped with performance enhancing substances and practices, but they were also entering “abnormal” women, with the goal of dominating the medal podium in the Olympic Games (18, 19). “Red “Wolves” in skirts” American journalist, Frank True, called them in 1966, summing up well what we can see as “Western” abjection in the face of the Soviet challenge to the patriarchal myth of female fragility. “If the Commies hadn”t been guilty of substituting men for women in the first place, the new rule of the IAAF wouldn”t have been necessary” (20). In their uncritical translation of contemporary Western values, regarding binary sex/gender boundaries, into universal sport policy, these concerns, and subsequently increased focus on sex verification, clearly illustrate the similarities to Fairchild's “abjection test.” The notion of natural sex/gender variation and the challenge it posed to the *status quo* was rejected (abjected) to confirm the established order of things. As long as female athletes with traditionally “masculine” features disrupt the perceived sex/gender norms, rule makers, and others, faced an internal conflict. The resulting conflict resolution was as follows: these muscular strong dominant athletes look more like males than females, therefore, these athletes must not be women. Perceived as “unnatural” and existing beyond the symbolic order, they are thus abjected. We can also see Jönsson's double standard at work, unlocking for women, but not men, the innate abjection potential of participation—and more specifically performance—in elite sport. Further, we can see in this the enduring echo of antiquated understanding of women's inferiority summed up well in Cynthia Freeland's feminist critique of Aristotle:

Aristotle says that the courage of a man lies in commanding, a woman's lies in obeying; that “matter yearns for form, as the female for the male and the ugly for the beautiful”; that women have fewer teeth than men; that a female is an incomplete male or ‘as it were, a deformity': which contributes only matter and not form to the generation of offspring; that in general ‘a woman is perhaps an inferior being’; that female characters in a tragedy will be inappropriate if they are too brave or too clever” (21)^19^

The inability of Brundage, and many others, to admit to the possibility of “masculine” looking women led directly to the institution of sex/gender verification and framed the debate as it continues to this day. Hence the medical/sport scientists have not only retained authority to determine who is allowed in the woman's sport category, often based on a medicalized sex-binary; but also the power to define what counts as “unfair advantage” in sport requiring the protection of the women's sport category, as was their responsibility in the realm of doping.

## 6. Protection of women's sport: transgender and gender diverse (TGD) athletes

This abjection of women relative to men is fundamental to our argument that Jönsson's double standard—and therefore abjection bias—both sustains, and is itself sustained, by the abjection of women simply because they are not men, and hence pose an enduring threat to normative maleness. This is in line with our definition of the abject as that which causes existential crisis by its non-conformity with the perceived *status quo*. But women athletes face a double abjection process; first, because they are not men, and second, because they are not normal women either.

The abjection process in the discussion of the protection of the women's sport category becomes even more complicated for transgender and gender diverse (TGD) individuals. We see a separate source of abjection in the transgression of the boundary between the traditional male versus non-male (*viz.* “female”) sex/gender binary. Thus, the determination of who is, and who is not, a woman athlete is simultaneously central to the binary differentiation of sex/gender both as a means of abjection of woman relative to normative “man,” but also the abjection of TGD individuals in the process of protecting normative “woman” against threats to the binary's symbolic order. Kimberlé Crenshaw's theory of intersectionality is clearly applicable (22) TGD athletes, particularly when seeking to compete in the women's category face abjection uniquely because they inhabit an identity that is—in the traditionally normative sense—simultaneously not a woman (non-biological sex female)-and not a man (-non-socio-cultural man).

While testosterone thresholds, which can be considered an outgrowth of anti-doping sciences, are currently the primary method to determine eligibility to compete in the women's category in many sports, the IOC and World Athletics have historically used biology and, in particular, chromosomes (female sex biologically determined by sex chromosomes XX). The eligibility criteria for fair play are, for the most part, about biological advantages. They have argued that the women's sport category was designed to protect those with a biological disadvantage (XX) in many sports and that sport sciences continue to demonstrate these facts (*e.g.*, world records in many sports, oxygen uptake, lung volume, muscle mass, *etc.* comparing men and women). The tested/exclusive category exists to protect women athletes who are deemed “naturally” disadvantaged from the competitive advantage of men and “unnatural” women. Currently, a significant issue is that there is no category for “legally determined women” (non biological females that have been recognized as women by the laws of their country) with naturally occurring above-average testosterone levels. Two related issues are: a) that of intersex individuals, whose biological sex includes both male and female; and b) that of retained “male advantage” despite pharmacological intervention to reduce testosterone levels in transgender individuals who have gone through male puberty and subsequently transitioned to become female.

Although they do not use the term “abjection,” Antoine Rajkovic and colleagues explain well the link between TGD individuals and what we would argue, includes abjection. They identify main ideas such as the “pathologization” of TGD individuals because of “sexual deviation” as well as the terrible drive in many times and places for removal of TGD individuals through “treatment” or even “eugenics” (23).^20^

Along with the challenge presented by intersexuality and sex variation, the question of transgender athlete participation has become—as well as doping—one of sport's most significant, and divisive, issues. This seems applicable to the question of TGD participation in sport. On the one hand, TGD athletes are seeking to participate within the standard model available (e.g., intersex and trans women wish to participate in the protected exclusive category “women”). This status quo in the rules and practices of sport that, where sex segregation rules exist, women compete with women, men with men primarily for reasons of fairness. Yet, there are two questions that tend to arise. These are distinct questions, although they are connected and sometimes conflated.

First, are TGD individuals who identify as “women” actually women athletes for the purposes of sport competition? And, who gets to decide? Legally in some places and instances, the answer is, yes. This has become a political question especially in socially conservative-leaning jurisdictions where the question of the legitimacy of transgender identity and gender reassignment are hotly debated with the authorities frequently adopting that stance that trans women are different from “natural” women—sometimes called “natal women”. This, of course, opens the door to abjection bias and falling into the now familiar trap of complacency in the face of Jönsson's double standard as well as complicity in the framing of policy by means of Fairchild's “abjection test”.

Mizuhu Takemura, in “Gender verification issues in women's competitive sports: An ethical critique of the IAAF DSD regulation,” reviews the work of Camporesi and McNamee, “On the eligibility of female athletes with hyperandrogenism to compete: athleticism, medicalization and testosterone” (24). Takemura argues that these authors thoroughly examined the ethical problems inherent in the hyperandrogenism regulations of IAAF through the cases of Dutee Chand and Caster Semenya, focusing on the Camporesi and McNamee's question: “For the purpose of international competition, how ought one to define femaleness or womanhood?” (24). Although agreeing with Camporesi and McNamee that the gender distinction based on the steroid values, as adopted by IAAF and IOC, falls into the trap of medical reductionism, Takemura rejects their proposal that eligibility could be determined by legal recognition as a woman in one's home country. Takemura (2020) points out that although it may seem reasonable for anyone legally recognized as a woman to be able to compete as a female athlete, by using the legal definition of woman, the question of maintaining fair play remains, in that, how are we to determine how athletes with gender identity disorder should be dealt with in the context of fair competiton?^21^ In the case of the Olympic games, the sports competitions are international events, but legal procedures for a sex change differ among countries and some countries do not even legally acknowledge sex changes, so athletes from such countries would have no remedies (24). Thus, Takemura concludes that the simple legal answer is at least as limited as the simple biological answer. So, arguments that assume that “gender identity” is taken to be already subsumed under the category of “sex” have serious limitations for human rights purposes. However, one could argue that, even if one rejects the legal status argument, gender identity could be positioned within interpretations of the *Olympic Charter* under the sixth principle of “or other status” and under protections against discrimination on the basis of “birth.” But it still remains the case that the rules designed to protect the women's category of sport, were designed for biological females which were deemed traditionally “normal” women and disadvantaged in competition in sport against biological males.

Thus, the second major question that must be addressed is: Are TGD individuals, who wish to compete in “women's” sport, unfairly advantaged relative to those who were born with “normal” XX chromosomes and assigned the gender “female” at birth? There is a growing body of scientific evidence showing that there is likely a physiological advantage (25). Whether and to what extent that advantage can, and more importantly, should, be pharmacologically mitigated is an ongoing debate.^22^ Fundamental to both questions is the lack of adequate competition space within sport's current sex-binary (women's sport on one hand and men's sport on the other) for women beyond the traditional weak-woman versus strong-man paradigm.

International sport bodies have moved away from direct means of sex-testing or “gender verification” (notably chromosomal) in recent years due to legal scrutiny of what has been challenged as an overly invasive and discriminatory practice. This move has meant that new methods of proving eligibility for participation in the protected “women's” category have had to be adopted. The IOC itself has attempted to strike a middle path that balances the traditional structure, in line with a recent ruling by the Court of Arbitration for Sport that “it is reasonable and proportionate to divide athletes into male and female categories” (26), while at the same time placing the responsibility of determining and enforcing qualification criteria on individual sports (27). and/or the laws of individual countries.

As identified by Takemura (24), a major complication with this approach is the international (and in some cases such as the United States, intra-national) patchwork of legal recognition of who is and is not a “woman”. Legal status can also be at odds with sports bodies who test levels of blood serum testosterone for qualification for inclusion in the women's category. Thus, we see the perplexing situation of “legally determined women” who are deemed ineligible and banned from competition due to high testosterone levels. Some have argued that from a human rights perspective, the default should be inclusion and not exclusion, particularly with regard to legally determined women with naturally occurring high testosterone levels. It has been argued (28) that this would require “doping down” to compete in the women's category. Anti-doping sciences would be required to test for evidence of this process. “Doping down” can be just as bad morally, as “doping up”. Caster Semenya lost her appeal to the Swiss Supreme Court to compete in the 800 metres against the World Athletics (formerly IAAF) regulations, passed in 2018, targeting intersex athletes who were born with both X and Y chromosomes (the traditional biological male sex pattern), and much higher levels of testosterone than the average biological female range. It is important to note that World Athletics did acknowledge that these regulations are discriminatory, but “do not exceed what is necessary in order to achieve equality of opportunity between male and female athletes, and are therefore proportionate” (29) to try to preserve fair play in the protected women's sport category.

Semenya's open defiance to the World Athletics policies and CAS ruling and refusing to “dope down” to comply with these regulations so she can compete in the women's category for the 800 meters, challenges Jonsson's double standard by reminding us of our common humanity. “I am very disappointed by this ruling,” she stated in response to a dismissal of her appeal of the CAS decision to the Swiss Federal Tribunal, “but refuse to let World Athletics drug me or stop me from being who I am […] endangering our health solely because of our natural abilities puts World Athletics on the wrong side of history” (30). This echos a statement by international advocacy group, Human Rights Watch, claiming the regulations amount to “policing of women’s bodies on the basis of arbitrary definitions of femininity and racial stereotypes” (31). The courts have ruled that it is a question of protecting the competitively disadvantaged traditional biological XX chromosomal women athletes. So, on the one hand, no qualifying athlete should have to “dope down” (or “dope up”) to compete. On the other hand, as Doriane Lambelet Coleman, former elite 800 meter runner and law professor claims, the Swiss Court ruling recognizes that “sex equality in competitive sport is a legitimate goal” and that “separating athletes in competition by biological sex traits is the only way to achieve this goal, given the physical advantages associated with male puberty and testosterone levels in the male range” (32).

There is no doubt that the debate will continue to be intense on this pressing issue, as it will be regarding doping, and that the anti-doping sciences will continue to be involved. Ultimately, however, abjection bias must itself be abjected if we hope to achieve meaningful and lasting progress that respects the human rights of all. In this, we must be willing to explore solutions beyond the *status quo* to ensure that sport is as inclusive as possible *and* as fair as possible.

## 7. Conclusion

In this paper we have argued that the concept of “abjection,” can help us to understand: i) the more extreme negative reaction that arises from seeing athletes who dope; ii) the even stronger reaction when it is female athletes who are doping; iii) that ii) is also historically tied to the conflation of doping and sex verification; and iv) that anti-doping sciences, in part, have been utilized, and still are, by sport administrators to help to maintain the perceived status quo.

Starting with a definition of the “abject,” as that which existentially challenges perceptions of symbolic order, we introduced the idea of “abjection bias” to conceptualize the perpetuation of attitudes to the “unnatural” that can occur in the absence abjection awareness. Through an examination of previous approaches to abjection in sport, we identified and rejected “Fairchild's abjection test” as an acceptable policy making tool. We acknowledged the role of “Jönsson's double standard” in explaining how individual and societal abjection bias leads to unequal perceptions of members of different groups, namely people with disability, women, and TGD individuals. Furthermore, we introduce the phrase “abjection potential” to clarify Jönsson's idea that elite competition carries with it an innate abject quality, whose realization depends on the identity of the individual or group in question. Furthermore, we suggest that these ideas open the door to a new theory of “intersectional abjection.” A brief history of these problems and the protection of the women's category in sport highlighted the connections and conflation between aspects of sex/gender verification and anti-doping sciences. It remains a very contentious issue, and just because we are able to identify abjection, it does not solve the problem of fairness, but it is important to understand some of the conceptual underpinning in order to make progress on it.

By understanding the historical role of medical and sport science both in identifying the sex/gender questions as a “problem” of “unnaturalness,” and the power granted these fields to determine appropriate “solutions,” we begin to grasp the conceptual and real link between doping and sex/gender in sport. We can also see the role of abjection bias in the development and implementation of policies and procedures aimed at the protection both of “clean” sport and “women’s” sport. It is important to note that much of the conceptual discussion on the topic of gender and doping has been tied to sport science and biological premises and paradigms, which, as we have shown, can be subject to abjection bias. The clarity gained from understanding this reasoning and the role of abjection and abjection bias can help shed light on current policy decision-making in relation to the protection of women's sport.

## Data Availability

Publicly available datasets were analyzed in this study. Further inquiries can be directed to the corresponding author.

## Footnotes

^1^Translated into English in 1982 in part by John Lechte (first chapter only as “Approaching Abjection”, *Oxford Literary Review*, 1982, Vol. 5, No. ½ (1982), pp. 125-149) and that same year in full by Leon S. Roudiez (*Powers of Horror: An Essay on Abjection*, Columbia University Press, 1982). We use the Roudiez translation throughout unless otherwise indicated.

^2^Like many late 20^th^ century European philosophers, Kristeva and her ideas have faced increasing scrutiny. While criticisms of the theorist focus on her supporting “a Eurocentric, colonial and orientalist perspective” (James 2021 … James, Julie. “Refusing Abjection: Transphobia and Trans Youth Survivance”. *Feminist Theory*, 2021. Vol. 22(1) 109-127 … see also Spivak, Gayatri Chakravorty. *French Feminism in an International Frame*. 1981), abjection theory itself has also been challenged on a more structural basis. Of particular concern is Winfried Menninghous’ 2001 critique that Kristeva has not provided a suitably robust theoretical basis upon which the concept of abjection can be explored (Menninghous, Winfried. *Disgust: Theory and History of a Strong Sensation*. Trans. Howard Eiland and Joe Golb. 2003. State University of New York Press. … original German publication 2001). Fortunately, re-readings of Kristeva offered by Katherine J. Goodnow (Goodnow, K.J. *Kristeva in Focus*. 2010. Oxford: Berghahn Books) and Robbie Duschinsky (Duschinsky, Robbie. “Abjection and Self-Identity: Towards a Revised Account of Purity and Impurity”. *Sociological Review*. University of Cambridge. Jan. 2013. 61: 709-727) offer a way forward with particular focus on the notions of “purity” and “impurity”, both central to abjection theory.

^3^Here we are to understand something like Jacques Lacan’s “symbolic” order, which effectively masks the underlying and indiscernible “real”. As Shuli Barzilai points out: “Throughout her theoretical writings, Julia Kristeva calls into question the privileged position of the symbolic order in Jacques Lacan’s teaching and clinical practice” (Barzilai 1991: 294). Shuli Barzilai (2020), Borders of Language: Kristeva’s Critique of Lacan, *Publicatoins of the Modern Languae Association of America, 106* (2). 294-305). DOI: https://doi.org/10.2307/462664

^4^From Latin *ab*- (off, away from) and *iacere* (to throw, impel)

^5^Indeed, “situate” may be misleading since someone cannot exactly be said to be placed into a no-place. It may be more correct to say, “exiled” from place all together. The abject as exile is an appealing notion especially given the connotation of being driven out, cast away (a castaway, abject) from the Subject-self and the ordered world.

^6^A literal *utopia*=“no place” *vs. eutopia*=“good place”, a pun due to the similar pronunciation of these Greek words.

^7^It is important here to clarify that challenging the logical validity of the ‘unnaturalness argument’ on the grounds that it is rooted in bias does not necessarily invalidate everything that it has been used to defend except where claims made under the unnaturalness argument are indefensible by means of more robust argumentation.

^8^There is a seemingly endless range of what exactly might be deemed ‘unnatural’ when using this argument with regard to doping. While Fairchild points to the method of drug delivery, other aspects might include from the motivation for its use (*e.g.* gaining an unfair advantage) as well as the resulting physical changes (*e.g.* gains in strength, speed, stamina that is more than normal for a human) and even undesirable side-effects of drug use (*e.g.* negative impacts on sexual function as can be the case with anabolic steroids). For women especially, the physical transformation that replaces traditional ’female’ qualities with ’male’ ones (*e.g.* gains in musculature) are frequently cited as aspects of an ’unnaturalness argument’ against the use of drugs in sport.

^9^Inversely, the existence of individual, group or even systemic abjection bias might fertilize the ground in which the unnaturalness argument can take root and flourish.

^10^It is important to note that this theme is not always viewed negatively, for example, transhumanists believe that the human race can evolve beyond its limitations by mean of science and technology with desirable superhuman properties that should help to improve the human condition.

^11^The official abjection of Johnson seems to have served a clear purpose: the re-establishment of symbolic order. If restoring perceptions of order is the goal, then only the tallest poppies need be cut down as an example to the rest.

^12^In some sense, officially sanctioned or directed abjection represents an Orwellesque ‘group-think’ in the abjectification process. The role of authority in what might be termed ‘official abjection ’also begs the question of whether and to what extent the consuming flame of abjection bias might be stoked, steered or perhaps even stamped out through official action.

^13^A big problem with Fairchild’s position is that while abjection bias gives rise to a tendency to abject that which existentially challenges the *status quo*, it does not necessarily follow that the *status quo* is morally or ethically justifiable. *e.g.* homosexuals at many times historically and even today in many places. It is unacceptable that we uncritically accept this reaction—much less allow it to direct policy. Such a sense of abjection in itself can be used to justify any number of mistreatments visited upon non-conforming individuals or communities.

^14^It is interesting to note that while the majority of competitors in that 100m Olympic final race would at various points afterwards face doping sanctions themselves or have since admitted to doping at those Games. Yet at those Games and in that situation, only Johnson’s drug use was officially confirmed; the extent of his abjection, therefore, seems to have been in part a means of restoring public confidence in the sport establishment’s ability to enforce the rules and ensure ’clean’ sport. In other words, abjection bias at work in the heavy handed response to a visible instance of non-conformity while turning of a blind eye on a wider doping problem and don‘t-ask-don‘t-tell culture that perpetuated the symbolic order.

^15^Hyphenated or contracted descriptive terms like “para-athlete”, “para-sport” and even “Paralympian” emphasize the distinction between normative sport as an able-bodied domain. “Para” is a prefix from Ancient Greek meaning something “alongside”, but also “beyond”, which positions (dis)abled athletes as something other than normal and unnatural, especially in terms of the technological and pharmacological adaptations that allow for participation in elite sport.

^16^Although unrelated to his sporting accomplishments, in his life “after” sport, in retirement Pistorius was convicted for homicide in the shooting death of his girlfriend. It would be interesting to investigate the role of abjection and his status as a Paralympian in media and legal portrayals of Pistorius during and after the trial.

^17^I appears that the concept of ‘intersectional abjection’ has been mentioned (but not elaborated upon) only a few times: in the context of literary studies (Lee, 2014), and decolonisation (Mir, 2018; Padilla, 2021). The authors of the current paper believe this idea has potential to be more fully developed in and beyond the sport context. (Seulghee Lee (2014),”’Other Lovings’: Abjection, Love Bonds, and the Queering of Race,” UC Berkeley Electronic Thesis and Dissertations. https://escholarship.org/uc/item/0mw1p6xm); (Carolta Mir (2018), ”Dossier for Critical Preservation and Re-use of Casa del Mutilato”, Decolonizing Architecture Advanced Course, Palermo, June 2018, https://www.daas.academy/wp-content/uploads/2020/01/10QueeringFascist_compressed.pdf); (Alexis Padilla (2021), ”Decoloniality, Embodiment and Othering Emotionality: Decoding and Countering the Inter-Imperialist Foundations of Intersectional Abjection,” Revista Latinoamericana de Estudios sobre Cuerpos, Emociones y Sociedad. N°37. Año 13. Diciembre 2021-Marzo 2022. Argentina. ISSN 1852-8759. pp. 89-99. https://dialnet.unirioja.es/servlet/articulo?codigo=8238041)

^18^Brundage’s use of the word ‘transformed’ is interesting for multiple reasons. First is the more recent use of the same prefix ‘trans’ as shorthand for ‘transgender’ or someone who has ‘transitioned’ from one gender to another (often by means of pharmaceuticals and/or surgery). It also connects us back to the ‘Frankenstein Factor’ and the oft-feared role of science in superseding the ‘natural’ order of things and hence raising the specter of abjection.

^19^Plato is a rare example of challenge to this prevailing view as pointed out in Schneider 2000.

^20^These are extreme forms of what in our paper might be called “radical exclusion” as in elimination, thus an ultimate form of abjection.

^21^Even medicalized terminology such as ’disorder’ implies ‘unnatural’ and thus contributes to abjection bias against those not conforming to the established ‘natural’ order.

^22^Schneider 2020 has argued that ‘doping down’ is as morally suspect as ‘doping up.’
